# Mother And late Preterm Lactation Study (MAPLeS): a randomised controlled trial testing the use of a breastfeeding meditation by mothers of late preterm infants on maternal psychological state, breast milk composition and volume, and infant behaviour and growth

**DOI:** 10.1186/s13063-020-4225-3

**Published:** 2020-04-07

**Authors:** Sarah Dib, Jonathan C. K. Wells, Mary Fewtrell

**Affiliations:** UCL Great Ormond Street Institute of Child Health, University College London, London, UK

**Keywords:** Preterm infants, Late preterm infants, Breastfeeding, Breast milk, Maternal stress, Infant growth, Infant behaviour, Meditation, Relaxation therapy, Oxytocin

## Abstract

**Background:**

Late preterm infants suffer from more complications and are less likely to be breastfed compared to term infants and their mothers experience higher levels of stress than mothers with term infants. The physiological or hormonal responses that influence milk ejection, milk production, and/or maternal behaviour are possible mechanisms by which maternal distress could negatively influence breastfeeding success. Maternal mood might also affect infant behaviour (feeding, sleeping, and crying) through changes in milk volume and composition, and consequently breastfeeding success and infant growth. Previous research, using relaxation therapy in 64 Malaysian first-time mothers breastfeeding their full-term infants, demonstrated that the therapy was effective in reducing maternal stress and improving infant growth. We hypothesise that expected benefits are even greater in a more vulnerable population where additional breastfeeding support is especially needed, such as in mothers of late preterm infants.

**Methods/design:**

This protocol describes our randomised controlled trial that tests whether a breastfeeding meditation audio reduces maternal stress in mothers of late preterm infants in London. Home visits will be conducted at 2–3 and 6–8 weeks post-delivery. Participants will be randomised to a control group or an intervention group, where mothers will be asked to listen to a meditation tape on a daily basis while breastfeeding. The main outcomes of the intervention will be maternal stress markers and infant weight Z-score. Potential mediators will be the secondary outcomes and include breast milk macronutrient and hormone levels (ghrelin, leptin, cortisol, and adiponectin), milk volume assessed by 48-h test-weighing, and maternal engagement with the infant. Infant behaviour, including crying and sleeping, and infant appetite will be evaluated. Data about other mediators such as maternal perception of milk supply and salivary oxytocin will be collected.

**Discussion:**

We hypothesise that the use of the breastfeeding meditation will reduce maternal stress and consequently improve infant growth mediated by changes in milk composition and volume and maternal behaviour. This study will allow us to understand the mother–infant factors that influence breastfeeding in late preterm infants and potentially identify a method that could improve mother, infant, and breastfeeding outcomes.

**Trial registration:**

ClinicalTrials.gov, NCT03791749. Registered 1 January 2019.

## Background

Nutrition during infancy has the potential to offer short- and long-term benefits through ‘programming’ during this critical window of growth [1]. Breast milk is an unparalleled source of nutrition that can also provide protective benefits for lactating mothers [2]. However, the percentage of women meeting the recommendations for breastfeeding duration, set forth by the World Health Organisation, is below target levels. The reasons for this are multiple and include the challenges that women face that hinder breastfeeding success, such as not receiving enough support. Interventions are therefore needed to support breastfeeding mothers to improve maternal, breastfeeding, and infant outcomes.

Breastfeeding also involves signalling between the mother and the offspring [3, 4]. Early-life environmental signals have the capability of modulating the development of the infant, leading to an adult physiology and metabolism, and consequently disease risk, that is influenced by those early-life signals [5]. The mother is the main source of these signals for young animals and, in Eutherian mammals, regulates the growth of her offspring through placental nutrition during gestation and through lactation post-delivery [6]. Maternal nutritional status, disease history, and social status might determine the signals the mother sends to her infant [6, 7]. Impaired maternal psychological state could also be a factor influencing the type of signals the mother is transferring to her infant. This could be in the form of altered production of breast milk and certain constituents of breast milk (macronutrients or hormones) and/or reduced nurturing behaviours (touching, holding, sensitivity to cues). Both these physical and physiological trade-offs may then alter infant development and behaviour accordingly [8]. Therefore, it is important to optimise the signals the mother is sending to her infant.

Other trade-offs between maternal investment and infant development may exist. During pregnancy and the postpartum period, many mothers experience cognitive changes including forgetfulness and poor concentration, colloquially known as ‘baby brain’ [9, 10]. These changes can be attributed to the hormonal changes mothers go through during these periods, namely changes in estradiol, progesterone, prolactin, oxytocin, and cortisol. It was previously shown that cortisol was associated in an inverted U-function with verbal recall scores and spatial abilities and that lower levels of cortisol were correlated with better attention scores [9]. Recently, significant reductions in grey matter involved in social cognition were found from pre- to post-pregnancy in first-time mothers, which might play a role in postnatal caregiving behaviours and to enhanced maternal–infant relationships [11]. Therefore, breastfeeding-associated hormones may influence cognitive function, facilitate adaptive brain restructuring, and improve maternal caregiving behaviours. Similarly, reducing maternal stress and thus cortisol levels might produce similar effects.

Late preterm infants (LPI), defined as infants born from 34 0/7 to 36 6/7 weeks, constitute 74% of all preterm births and are the fastest growing subset of neonates [12]. Compared to term infants, LPI have a significantly higher risk of morbidity, including jaundice, hypoglycaemia, temperature instability, and respiratory distress [13, 14]. The last 6 weeks of gestation represent a critical period for brain development; therefore, LPI may have an increased risk of behavioural and emotional problems and inferior academic performance later in life [15–18].

Breastfeeding difficulties are the leading complication experienced by LPI [19]. They are less likely to be breastfed, which could be due to infant- and maternal-related barriers. Infant barriers include poor muscle tone and neurodevelopmental immaturity, which may interfere with effective sucking patterns and the ability to give hunger cues. LPI are also more likely to experience rapid fatigue during feeding, lower stamina, fewer awake periods, and reduced effort to stimulate and empty the breast, consequently leading to poor milk production and inadequate intake [19–21]. Maternal barriers to breastfeeding may include various factors associated with preterm birth, such as caesarean delivery, obesity, multiple births, or maternal psychological distress [19]. Mothers of LPI may also experience higher levels of anxiety, depression, and stress, each of which is an independent risk factor for breastfeeding failure in this population [22]. Lastly, some of the commonly cited breastfeeding challenges for mothers of LPI include perceived insufficient milk supply and inadequate support by health care providers post-discharge [23, 24].

This is important because premature infants, including LPI, extract the greatest benefit from receiving breast milk [25–28]. Due to their developmental immaturity and increased susceptibility to inflammation, oxidative stress, and infections, breastfeeding and the constituents of breast milk are particularly beneficial for this group [29, 30]. For instance, in a large cohort of 1130 late and moderately preterm infants, not receiving breast milk at hospital discharge was an independent risk factor for moderate/severe cognitive impairment at 2 years of age [31].

Previously, two RCTs used a relaxation tape as a form of relaxation therapy and found that it increased milk production in mothers of preterm infants [32, 33]. These studies have some methodological limitations that ought to be addressed in future RCTs. Firstly, in one study, milk volume at baseline was not measured and milk production was assessed from a single breast expression session at the end of the study. In both studies, it was unclear whether the milk collection procedure was standardised for time of last feed, time of day, or lactational stage; all of which may influence the volume of milk available for expression. Lastly, neither study reported the exclusivity of breastfeeding, which can influence milk yield. Another RCT investigated the use of a guided imagery meditation tape for breastfeeding mothers of healthy term infants in Malaysia [34]. The therapy was effective in reducing maternal stress and increasing infant weight gain and sleep duration. This research showed that relaxation therapy has benefits for mothers and infants that belong to a low-risk group (high socioeconomic status, full-term infant, healthy mother, high family support, practicing traditional confinement period). Therefore, the benefits might be even greater in higher risk groups, such as mothers of premature infants.

This study will examine the psychological, behavioural, anthropological, and physiological aspects of lactation in breastfeeding mothers of LPI. The primary aim is to determine the effect of a relaxation therapy on maternal stress and infant weight gain. Secondary outcomes of the intervention include breast milk composition (macronutrients, human milk oligosaccharides (HMO), ghrelin, leptin, cortisol, adiponectin), breast milk volume, infant appetite and behaviour, maternal engagement, maternal cognitive function, and maternal salivary cortisol and oxytocin. These secondary outcomes will help address the potential mechanisms by which maternal stress could affect infant growth and ascertain whether there are maternal costs as well as infant benefits.

## Methods/design

### Study design

**Fig. 1:**
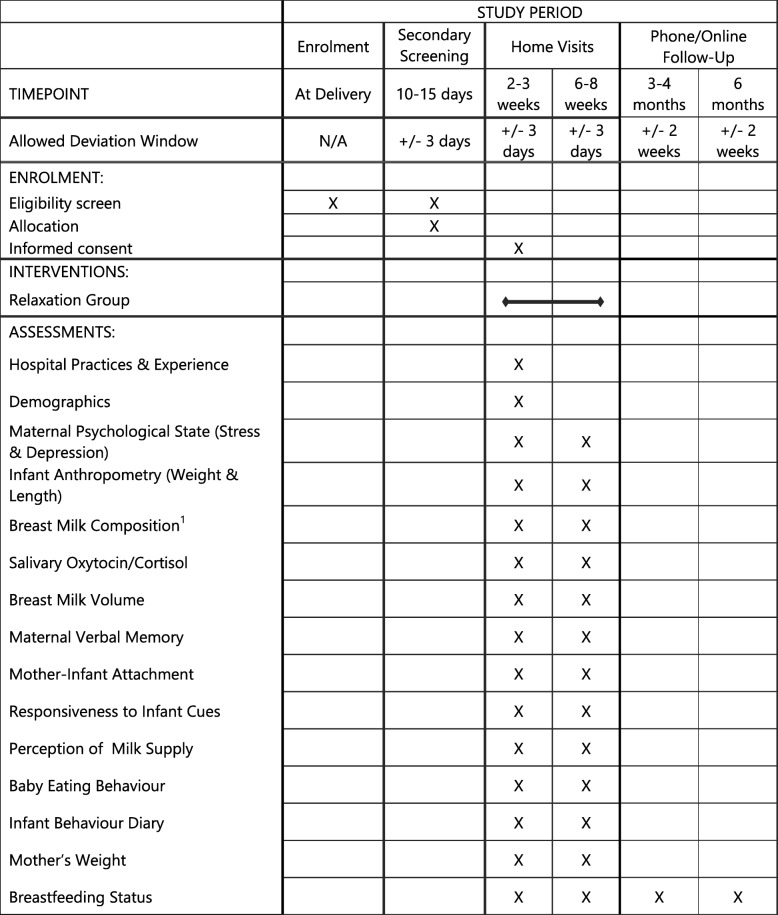
Standard Protocol Items: Recommendations for Interventional Trials (SPIRIT) figure; schedule of enrolment, interventions, and assessments. ^1^Breast milk samples will be collected in a standardised procedure and will be analysed for macronutrient, human milk oligosaccharide, ghrelin, leptin, adiponectin, and cortisol content

**Fig. 2:**
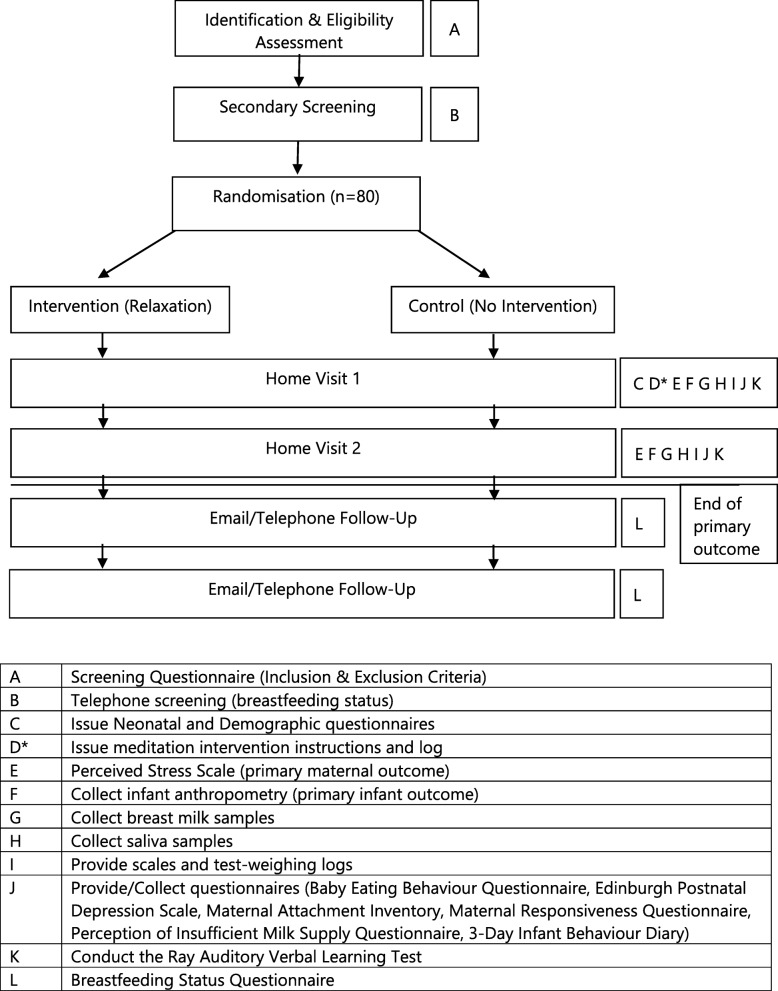
Study flow chart. *Intervention group only

The Mother and late Preterm Lactation Study (MAPLeS) is an RCT using a breastfeeding meditation for breastfeeding mothers of LPI. Participants will be identified from multiple hospitals with the capacity to care for LPI in London, UK. Eligible mothers will be randomly allocated to either the intervention group, where they will be asked to listen to the relaxation therapy, or to the control group where no intervention is given. The first home visit will be conducted at 2–3 weeks post-delivery, when treatment will commence. At this time point, it is likely that LPI would be discharged from the hospital and that breastfeeding would be somewhat established. One of the main aims of the study is to investigate mother–infant signalling through breastfeeding; therefore, it is important to target women who are more likely to be breastfeeding for the duration of the study. The second home visit will be at 6–8 weeks post-delivery for follow-up. At both time points, questionnaires, breast milk and saliva samples, and anthropometric measurements will be collected. We will also follow-up at 3 and 6 months post-delivery by email or phone to assess breastfeeding status. The SPIRIT figure and study design flow chart are shown in Figs.1 and 2.

### Hypotheses

#### Primary hypotheses

1. Mothers in the intervention group, using the relaxation therapy from week 2–3 post-delivery, will have a higher reduction in maternal stress score (*primary maternal outcome*) by week 6–8 post-delivery compared to mothers in the control group.
2. Infants in the intervention group will have a higher gain in weight Z-score (*primary infant outcome*) by week 6–8 post-delivery compared to infants in the control group.

#### Secondary hypotheses

1. The use of relaxation therapy at week 2 post-delivery will result by 6–8 weeks post-delivery in:
  Increased fat and energy content of breast milk
  Higher concentration of HMOs, ghrelin, leptin, and adiponectin in breast milk and lower cortisol
  Increased milk volume
  Higher concentration of salivary oxytocin
  Reduced time spent crying and increased duration of sleep by the infant
  Higher prevalence of exclusive breastfeeding
  Better verbal memory scores
2. Ghrelin, leptin, and adiponectin concentrations in breast milk are associated with:
  Infant behaviour (time spent crying and duration of sleep) and appetite traits
  Infant weight Z-score
  Maternal BMI, pregnancy weight gain, and pre-pregnancy BMI

### Participants

Breastfeeding mothers of LPI will be eligible to participate in this study. The specific inclusion and exclusion criteria are detailed in Table 1.

**Table 1:** Inclusion and exclusion criteria

Screening	Inclusion Criteria	Exclusion Criteria
Primary Screening	Infant born late preterm (34–37 weeks) & medically stable	Infant born < 34 weeks or > 37 weeks and/or requires hospitalisation for more than 2 weeks
	Singleton pregnancy	Multiple pregnancy
At Recruitment	Intending to breastfeed for at least 6 weeks	Intending to breastfeed for less than 6 weeks
	Mother and infant free of serious illness that may impact breastfeeding	Mother and/or infant have serious illness that interferes with breastfeeding
	Speaks and understands English	Does not understand or speak English
	Non-smoker	Mother currently smoking or intending to smoke while breastfeeding
	Based in London	Not based in London
	Mothers with no prior breast surgery	Mothers with prior breast surgery that affects breastfeeding
Secondary Screening At Phone Follow Up	Interested in participating further	Not interested or unable to participate further
	Still intending to breastfeed for at least 6 weeks	Intending to breastfeed for less than 6 weeks
	Breastfeeding at 2 weeks	Only providing formula

### Sample size

A power calculation was carried out to estimate sample size according to the following equation:

$$ \mathrm{N}=\frac{2F{\sigma}^2}{d^2}. $$

where N = sample size, *σ* = standard deviation, *d* = difference, and *F* = constant related to statistical power and confidence interval.

A previous similar study was able to detect significant and clinically important differences in weight Z-score (*σ* = 0.8, d = 0.63) and stress score (*σ* = 4.4, d = 3.51) in response to the same relaxation intervention (meditation audio) but in mothers of healthy term infants. Our sample of breastfeeding mothers of LPI might be more stressed and thus may respond less to the relaxation intervention. Therefore, a sample of 68 mothers (34 randomised to each group) is adequate to detect a three-point difference in stress score and a 0.55 weight Z-score difference significant at 5% with a power of 80%. This may be a challenging population and mothers may not complete the study measurements; therefore, accounting for that and drop-outs, we aim to recruit a sample of 80 mother–infant pairs.

### Research procedures

#### Recruitment (0–2 weeks post-delivery)

Participants will be identified mainly from the Royal Free Hospital, Barnet Hospital, and University College London Hospital. The study flyer will also be advertised on social media. The main researcher will provide interested mothers with participant information sheets and confirm their eligibility. Informed consent will be obtained at the first home visit.

#### Follow-up screening by telephone (1–2 days prior to first home visit due date)

Mothers will be contacted a few days before the first home visit to determine if they are still eligible to participate. They will be asked whether they are still breastfeeding and intending to continue breastfeeding for at least 6 weeks. If still eligible, the time of the first home visit will be confirmed and mothers will be reminded of the preparations required for the breast milk and saliva sample collection.

#### First home visit (HV1; 2–3 weeks post-delivery)

At the first home visit, saliva and breast milk samples will be collected using standardised methods. Anthropometric measurements including infant’s weight and length and mother’s weight will be taken. The main researcher (SD) will also conduct the Rey Auditory Verbal Learning Test (RAVLT) to assess the mother’s verbal memory. The Neonatal Questionnaire will be administered during the home visit to assess early hospital practices such as timing of breastfeeding initiation, skin-to-skin contact duration, type of delivery, and feeding practices at the hospital.

To reduce the burden of form filling on the participants, a study folder containing the following questionnaires will be left at the participant’s home to be filled at the mother’s convenience in the three subsequent days:

1. Perceived Stress Scale (PSS) and Edinburgh Postnatal Depression Scale (EPDS)
2. Perception of Insufficient Milk Questionnaire (PIMQ)
3. Maternal Attachment Inventory (MAI)
4. Maternal Responsiveness Questionnaire (MRQ)
5. Breastfeeding Status Questionnaire adapted from the CDC Infant Feeding Practices Study II Questionnaires
6. Baby Eating Behaviour Questionnaire (BEBQ)
7. 3-Day Infant Behaviour Diary
8. Demographic questionnaire

Finally, an infant weighing scale and log will be given to the participants to complete 48 h of test-weighing to assess average milk consumption.

#### Randomisation and blinding

After the first home visit, each mother will be randomly allocated to either the intervention group (relaxation therapy) or control group. Randomisation assignments will be prepared by a member of the research team, who will have no contact with the mothers, using a computer-generated list stratified for gestational age and parity. Assignments will be stored in sealed opaque envelopes. The assignment will be revealed 3 days after the first home visit when the researcher will retrieve the infant weighing scale from the participant and provide mothers in the intervention group with the meditation tape and meditation use log. To avoid contamination between the intervention and control group, mothers will not be informed about this process until the end of the study, when they will all be offered the meditation.

#### Second home visit (6–8 weeks post-delivery)

The same research procedures will be carried out at the second home visit and the mothers will be asked to complete the same questionnaires, except for the neonatal and demographic questionnaires.

Participants will be reimbursed (£30 voucher) for their time at the second home visit, which might encourage them to complete the follow-up.

#### Telephone/online follow-up (3 and 6 months post-delivery)

Breastfeeding success and intensity will be assessed using the Breastfeeding Status Questionnaire. Information about solid food introduction will also be collected.

### Intervention

Mothers in the intervention group will be given a breastfeeding meditation recording and instructed to listen to it during breastfeeding or pumping. The audio is based on a guided imagery meditation originally developed by Sheri Menelli in 2001 [35] and previously used in three RCTs [32–34]. The audio that will be used is 8 min in duration and has been adapted to suit the current population. Mothers will be encouraged to use it as many times as they would like, but at least once a day for the first 2 weeks starting at HV1 (2–3 weeks post-delivery). They will also be asked to keep track of the frequency and dates the meditation audio was used in the listening logs provided.

#### Pilot study

A within-subject pilot study was conducted at University College London to compare the effectiveness of five different relaxation interventions (guided-imagery meditation audio, music listening, relaxation lighting, combination of relaxation lighting and guided-imagery meditation, combination of relaxation lighting and music listening) and one control (silence/sitting) in 17 women of reproductive age. Subjective feelings of relaxation (ten-point scale), heart rate, systolic and diastolic blood pressure, and fingertip temperature were measured before and after each technique. Based on the results, meditation was selected as the most appropriate intervention for use with breastfeeding mothers of LPI.

### Control

Participants in the control group will receive no intervention and will be informed that the purpose of the study is to investigate the factors that influence breastfeeding in mothers of LPI and to study mother–infant signalling through breastfeeding. All participants will be informed of the randomisation process after completion of the trial and control mothers will be offered the intervention.

### Outcome measures

#### Primary outcome measures

##### Maternal stress

PSS will be used to assess maternal stress levels at both home visits; a ten-item scale, it is used to assess the extent to which someone’s life is seen as stressful and uncontrollable [36]. Each item is scored on a five-point scale (0–4) and thus the total could range from 0 to 40. Higher scores indicate higher stress. The PSS has been validated in English and has a Cronbach’s alpha of 0.85 [36]. It has also been widely used in studies, including three national surveys in the US [37] and a previous similar trial for breastfeeding women [34].

##### Infant weight

Weight will be measured using a digital infant weight scale (Seca, UK). Infants will be weighed naked and pre-feeding, if possible. Measurements will be taken two times, and the mean of the two values will be used to calculate weight Z-score for analysis.

#### Secondary outcomes

##### Breast milk composition

Breast milk samples will be collected for macronutrient and hormone analysis. Mothers will be asked to collect milk with the use of their electric or manual pump. If they do not own one, they will be provided with a manual pump (Philips AVENT, Philips, The Netherlands). Khan et al. showed that fat content varied significantly over a 24-h period, being highest at mid-morning, and was associated with the degree of breast fullness/emptying [38]. Therefore, participants will be asked to provide a full expression from a breast that has been emptied in the previous feeding session with at least 2-h wait. All home visits will take place in the morning and breast milk will be collected at 11:00 h ± 1.5 h. These measures will ensure that the sample provides the closest indication of nutrient per feed and reduces the between- and within-subject variability in milk composition. If possible, a volume of 15 ml will be transferred to 5 ml and 1.5 ml tubes, and the remaining milk in the collection bottle will be returned to the mother for feeding later/immediately. The samples will be transferred in a freezer bag with frozen ice packs from the participant’s home to − 80 °C storage until analysis. Macronutrients will be analysed using the MIRIS Human Milk Analyser (Miris, Sweden), while the hormones (ghrelin, leptin, adiponectin, cortisol) will be analysed using an enzyme-linked immunosorbent assay kit.

##### Breast milk supply

The volume of breast milk consumed over a period of 48 h will be determined using the test-weighing method. Mothers will be instructed to weigh their baby before and after each feeding using the electronic baby scale provided by the researcher. The participants will be asked to change the infant’s nappy before taking the first measurement. Measurements will be recorded by the mother on the test-weighing log provided, including the duration of the feed. Correction will be made for insensible water loss during the feed [39]. Several studies have shown that test-weighing before and after a feed, whether it is formula feeding or breastfeeding, is a valid method especially when using an electronic scale [40]. It was also shown to be useful in research, clinical, and home settings. A breastfeeding log will be provided, including the timings and durations of the feed, to help us estimate breast milk intake in case of missing weighings.

##### Infant length

Length and infant weight will be taken to measure infant growth. Recumbent length will be assessed using an infant length measuring mat. All infants will be placed in a supine position with their head against the headboard and the base of their feet against the vertical plate. The BMI will also be calculated (weight (kg)/length (m^2^)). Length and body mass index Z-scores will be calculated for analysis.

##### Infant behaviour

Infant eating behaviour and appetite will be assessed using the BEBQ. It is an 18-item parent-reported questionnaire that measures overall appetite and assesses four eating traits: ‘enjoyment of food’, ‘food responsiveness’, ‘slowness in eating’, and ‘satiety responsiveness’ [41]. These traits might be related to infant weight gain and might be altered with maternal psychological distress (possibly meditated through breast milk composition).

Infant crying and sleeping will be measured using a 3-day infant behaviour diary, which was previously validated against audiotapes and against actigraphy and showed good compliance [42]. The 72-h diary is divided into 15-min segments, where the mother will be asked to indicate whether the infant was crying, sleeping, feeding, being fussy, or was awake and content.

##### Mother–infant attachment

The MAI, consisting of 26 items, will be used to measure the attachment between each participant and her baby. Each statement is marked on a four-point Likert scale. The sum of the 26 questions will yield the total score, where higher scores indicate better attachment. It demonstrated a Cronbach’s alpha of 0.85 when used to measure maternal attachment with 4-week old infants [43].

##### Maternal depression

Maternal depression will be assessed using the EPDS, a ten-item self-administered scale [44]. Each item is scored on a four-point scale (0–3) with total scores ranging from 0 to 30. Higher scores indicate a higher intensity of symptoms experienced during the previous week. EPDS has been validated in English and has a Cronbach’s alpha of 0.86.

##### Cognitive function

Maternal verbal learning and memory will be evaluated using RAVLT to assess the ‘baby brain’ phenomenon. It consists of five recall trials (trials 1–5) using list A (15 noun word list) and one recall after an interference period (trial 6) using list B (another 15 noun word list). The outcomes of the test will include: immediate recall (sum of correct responses after first five trials) and verbal learning (difference in number of correct responses after trial 5 and trial 1) [45].

##### Perception of milk supply

PIMQ, a self-report, six-item scale, will assess maternal perception of insufficient milk supply [46]. The first item of the questionnaire will ask whether the participant believed she had sufficient milk to satisfy her infant (yes/no). This is followed by five questions relating to the reasons for this belief, measured on a ten-point Likert scale ranging from 0 (strongly disagree) to 10 (strongly agree). Higher scores indicate higher perception of having adequate milk. PIMQ showed good internal consistency with a Cronbach’s alpha of 0.70.

##### Maternal responsiveness

Maternal responsiveness to infant cues will be assessed using the MRQ. MRQ measures the extent to which mothers respond promptly to their infants across different situations [47].

##### Salivary hormones

Saliva samples for hormone analysis will be collected using the passive drool method where the mother will transfer 500 μl saliva into a 1.5 collection tube using a Saliva Collection Aid (Salivette, Sarstedt, Germany). Samples will be transferred in a freezer bag with frozen ice packs from the participant’s home to − 80 °C storage until analysis.

### Data management, monitoring, and dissemination

Identifiable data (hard copy) collected, including contact data, will be stored in locked filing cabinets at the Institute of Child Health separate from other study data. All data collection forms, questionnaires, and labels will include only the participant study ID number. These pseudo-anonymised forms will be stored in filling cabinets at University College London. Breast milk and saliva samples collected will be stored in a locked nutrition freezer unit. The results of the study will be published in peer-reviewed journals and will be presented at scientific conferences. No identifiable data will be published or presented to maintain the anonymity of the participants.

There are no arrangements for a formal data monitoring committee to be convened since the study is of short duration and the intervention is not considered to pose any risk.

### Statistical analysis

Data will be entered and analysed on SPSS v23.0. Kolmogorov-Smirnov test will be used and histograms and Q-Q plots will be assessed to test for normality. Results will be considered statistically significant at *p* < 0.05. The main analysis of the randomised controlled trial results will be an intention-to-treat analysis, but we will also be looking at whether there is a ‘dose–response’ effect between the use of the audio and the outcomes. For the primary outcomes, the differences in weight Z-score and PSS score between groups will be analysed using independent sample *t*-test, taking into account the baseline weight and stress scores (Supplementary Table 1). Similarly, for the secondary outcomes, differences between control and intervention in milk composition, milk supply, maternal cognitive function, maternal salivary cortisol/oxytocin, EPDS score, mother–infant attachment, and infant behaviour will be analysed using independent sample *t*-test. As a secondary analysis, the relationship between breast milk hormones and infant behaviour, growth and sex and maternal anthropometric measures will be analysed using univariate regression, while adjusting for confounding factors such as randomisation status, gestational age, and bottles of formula fed in the previous 7 days (Supplementary Table 1).

## Discussion

This study will allow us to observe causal relationships between maternal stress and infant growth and to investigate potential mediators between the two including breast milk composition and volume, maternal engagement, and infant behaviour. We will also examine whether reducing maternal stress levels, and thus maternal costs, could help improve both cognitive function and caregiving behaviours.

While the gold standard for estimating milk intake is the ‘dose-to-mother’ stable isotope technique, it will not be possible to use it with this sample. Mothers of LPI are likely to be expressing milk, to increase their supply or to provide top ups. They might also be providing formula top ups. In this trial, therefore, we will be asking the mothers to test-weigh their infants for 48 h and to log the amount of expressed breast milk or formula consumed at each feed.

If proven beneficial, the study will be able to highlight a simple accessible method to improve breastfeeding outcomes in a vulnerable population. It will also provide insight on mechanisms by which maternal stress can influence breastfeeding success and infant development and help in understanding how to optimise late preterm infant growth. We will also be able to identify some of the barriers and facilitators of breastfeeding a LPI.

### Trial status

The trial is in the recruitment phase and commenced February 1st 2019 and is expected to be completed March 1st 2020. The protocol was amended and approved on March 5th 2019 (version 3.0).

## Supplementary information

**Additional file 1: Supplementary Table 1.** Main hypotheses and statistical analysis plan.

## Data Availability

The datasets generated and/or analysed during the current study are not publicly available due to individual privacy concerns but are available from the corresponding author on reasonable request.

## Abbreviations

BEBQ: Baby Eating Behaviour Questionnaire
EPDS: Edinburgh Postnatal Depression Scale
HMO: Human milk oligosaccharides
LPI: Late preterm infants
MAI: Maternal Attachment Inventory
MAPLeS: Mother and late Preterm Lactation Study
PIMQ: Perceived Insufficient Milk Supply Questionnaire
RAVLT: Ray Auditory Verbal Learning Test
RCT: Randomised controlled trial

## References

[1] Lucas A (1998). Programming by early nutrition: an experimental approach. J Nutr.

[2] Victora CG (2016). Breastfeeding in the 21st century: epidemiology, mechanisms, and lifelong effect. Lancet.

[3] Wells JC (2003). Parent-offspring conflict theory, signaling of need, and weight gain in early life. Q Rev Biol.

[4] Wells J (2006). The role of cultural factors in human breastfeeding: adaptive behaviour or biopower. J Hum Ecol.

[5] Power ML, Schulkin J (2013). Maternal regulation of offspring development in mammals is an ancient adaptation tied to lactation(). Appl Transl Genom.

[6] Wells JC (2010). Maternal capital and the metabolic ghetto: an evolutionary perspective on the transgenerational basis of health inequalities. Am J Hum Biol.

[7] Marphatia AA (2016). Associations of gender inequality with child malnutrition and mortality across 96 countries. Glob Health Epidemiol Genom.

[8] Wells JCK (2019). Low maternal capital predicts life history trade-offs in daughters: why adverse outcomes cluster in individuals. Front Public Health.

[9] Henry JF, Sherwin BB (2012). Hormones and cognitive functioning during late pregnancy and postpartum: a longitudinal study. Behav Neurosci.

[10] Davies SJ (2018). Cognitive impairment during pregnancy: a meta-analysis. Med J Aust.

[11] Hoekzema E (2017). Pregnancy leads to long-lasting changes in human brain structure. Nat Neurosci.

[12] Davidoff MJ (2006). Changes in the gestational age distribution among U.S. singleton births: impact on rates of late preterm birth, 1992 to 2002. Semin Perinatol.

[13] Boyle EM (2015). Neonatal outcomes and delivery of care for infants born late preterm or moderately preterm: a prospective population-based study. Arch Dis Child Fetal Neonatal Ed.

[14] Kalyoncu O (2010). Neonatal morbidity and mortality of late-preterm babies. J Matern Fetal Neonatal Med.

[15] McGowan JE (2011). Early childhood development of late-preterm infants: a systematic review. Pediatrics.

[16] de Jong M, Verhoeven M, van Baar AL (2012). School outcome, cognitive functioning, and behaviour problems in moderate and late preterm children and adults: a review. Semin Fetal Neonatal Med.

[17] Shah P (2016). Developmental outcomes of late preterm infants from infancy to kindergarten. Pediatrics.

[18] MacKay DF (2010). Gestational age at delivery and special educational need: retrospective cohort study of 407,503 schoolchildren. PLoS Med.

[19] Radtke JV (2011). The paradox of breastfeeding-associated morbidity among late preterm infants. J Obstet Gynecol Neonatal Nurs.

[20] Darcy AE (2009). Complications of the late preterm infant. J Perinat Neonatal Nurs.

[21] Pados BF (2007). Safe transition to home: preparing the near-term infant for discharge. Newborn Infant Nurs Rev.

[22] Zanardo V (2011). Psychological distress and early lactation performance in mothers of late preterm infants. Early Hum Dev.

[23] Kair LR, Colaizy TT (2016). Breastfeeding continuation among late preterm infants: barriers, facilitators, and any association with NICU admission?. Hosp Pediatr.

[24] Kair LR (2015). The experience of breastfeeding the late preterm infant: a qualitative study. Breastfeed Med.

[25] Schanler RJ, Shulman RJ, Lau C (1999). Feeding strategies for premature infants: beneficial outcomes of feeding fortified human milk versus preterm formula. Pediatrics.

[26] Quigley M, Embleton ND, McGuire W. Formula versus donor breast milk for feeding preterm or low birth weight infants. Cochrane Database Syst Rev. 2018;(6):CD002971. 10.1002/14651858.CD002971.pub4. 10.1002/14651858.CD002971.pub4 PMC6513381 29926476

[27] Singhal A (2004). Breastmilk feeding and lipoprotein profile in adolescents born preterm: follow-up of a prospective randomised study. Lancet.

[28] Singhal A, Cole TJ, Lucas A (2001). Early nutrition in preterm infants and later blood pressure: two cohorts after randomised trials. Lancet.

[29] Ballard O, Morrow AL (2013). Human milk composition: nutrients and bioactive factors. Pediatr Clin N Am.

[30] Meier P (2013). Management of breastfeeding during and after the maternity hospitalization for late preterm infants. Clin Perinatol.

[31] Johnson S (2015). Neurodevelopmental outcomes following late and moderate prematurity: a population-based cohort study. Arch Dis Child Fetal Neonatal Ed.

[32] Feher SDK (1989). Increasing breast milk production for premature infants with a relaxation/imagery audiotape. Pediatrics.

[33] Keith DR, Weaver BS, Vogel RL (2012). The effect of music-based listening interventions on the volume, fat content, and caloric content of breast milk–produced by mothers of premature and critically ill infants. Adv Neonatal Care.

[34] Mohd Shukri NH (2019). Randomized controlled trial investigating the effects of a breastfeeding relaxation intervention on maternal psychological state, breast milk outcomes, and infant behavior and growth. Am J Clin Nutr.

[35] Menelli S. Breastfeeding meditation. Encinitas: White Heart Publishing; 2004.

[36] Cohen S, Kamarck T, Mermelstein R (1983). A global measure of perceived stress. J Health Soc Behav.

[37] Cohen S, Janicki-Deverts D (2012). Who’s stressed? Distributions of psychological stress in the United States in probability samples from 1983, 2006, and 2009. J Appl Soc Psychol.

[38] Khan S (2012). Variation in fat, lactose, and protein composition in breast milk over 24 hours: associations with infant feeding patterns. J Hum Lact.

[39] Wu PYK, Hodgman JE (1974). Insensible water loss in preterm infants: changes with postnatal development and non-ionizing radiant energy. Pediatrics.

[40] Scanlon KS (2002). Assessment of infant feeding: the validity of measuring milk intake. Nutr Rev.

[41] Llewellyn CH (2011). Development and factor structure of the Baby Eating Behaviour Questionnaire in the Gemini birth cohort. Appetite.

[42] Müller S, et al. Parental report of infant sleep behavior by electronic versus paper-and-pencil diaries, and their relationship to actigraphic sleep measurement. J Sleep Res. 2011;20(4):598–605. 10.1111/j.1365-2869.2011.00926.x 21707809

[43] Muller ME. A questionnaire to measure mother-to-infant attachment. J Nurs Meas. 1994;2(2):129–41. 7780768

[44] Cox JL, Holden JM, Sagovsky R. Detection of postnatal depression. Development of the 10-item Edinburgh Postnatal Depression Scale. Br J Psychiatry. 1987;150(6):782–6. 10.1192/bjp.150.6.782 3651732

[45] Estevez-Gonzalez A, et al. Rey verbal learning test is a useful tool for differential diagnosis in the preclinical phase of Alzheimer’s disease: comparison with mild cognitive impairment and normal aging. Int J Geriatr Psychiatry. 2003;18(11):1021-8. 10.1002/gps.1010 14618554

[46] McCarter-Spaulding DE, Kearney MH. Parenting self-efficacy and perception of insufficient breast milk. J Obstet Gynecol Neonatal Nurs. 2001;30(5):515-22. 10.1111/j.1552-6909.2001.tb01571.x 11572532

[47] Leerkes E, Qu J. The Maternal (Non) Responsiveness Questionnaire: initial factor structure and validation. Infant Child Dev. 2017;26(3):e1992. 10.1002/icd.1992 PMC5608276 28943807

