# Supplementary material for: Mother And late Preterm Lactation Study (MAPLeS): a randomised controlled trial testing the use of a breastfeeding meditation by mothers of late preterm infants on maternal psychological state, breast milk composition and volume, and infant behaviour and growth
Source: Trials. 2020 Apr 7;21:318. doi: 10.1186/s13063-020-4225-3 (PMC7137320; doi:10.1186/s13063-020-4225-3)
Supplement: Supplementary file 1 — Additional file 1: Supplementary Table 1. Main hypotheses and statistical analysis plan. [file 13063_2020_4225_MOESM1_ESM.docx]

Supplementary Table 1. Main Hypotheses and Statistical Analysis Plan

| **Main Hypotheses and Outcomes:** | **Primary Analysis** | **Potential confounding factors to be considered** |
| --- | --- | --- |
| **1.The use of relaxation therapy from 2-3 weeks post-delivery will result at 6-8 weeks post-delivery in:** |  |  |
| Reduced maternal stress scores (PSS Score) | T-test* | PSS score at HV1 |
| Higher infant weight gain (Weight Z-Score) | T-test* | Weight Z-score at HV1 |
| **2.The use of relaxation therapy from 2-3 weeks post-delivery will result at 6-8 weeks post-delivery in:** |  |  |
| Higher fat (g/dL) and energy (Kcal/dL) content of breast milk | T-test* | Fat and energy at HV1 |
| Higher concentration (ng/ml) of leptin, ghrelin and adiponectin, and lower concentration of cortisol (pg/ml) | T-test* | Hormones concentration at HV1 |
| Increased milk volume (ml) | T-test* | Milk volume at HV1 |
| Higher concentration of salivary oxytocin (pg/ml) | T-test* | Concentration of oxytocin at HV1 |
| Reduced time spent crying (min) and increased duration of sleep (min) by the infant | T-test* | Time spent crying and sleeping at HV1 |
| Better verbal memory scores (RAVLT score) | T-test* | Verbal memory scores at HV1 |
| **3.Ghrelin, leptin and adiponectin concentrations in breast milk are associated with:** |  |  |
| Time spent crying, duration of sleep and appetite traits | Univariate Regression | Infant weight, sex, gestational age, numbers of bottles fed in the last 7 days, times listened to intervention |
| Weight gain (Weight Z-Score) | Univariate Regression | Gestational age, infant sex, numbers of bottles fed in the last 7 days, times listened to intervention |
| Maternal BMI (kg/m^2^), pregnancy weight gain (kg), and pre-pregnancy BMI (kg/m^2^) | Univariate Regression | Times listened to the intervention |

*Independent sample t-test for differences between intervention and control group
